# Molecular docking insights of Nigella sativa compounds as potential antiviral inhibitory agents against the replication-machinery proteins VPg and RdRP in rabbit hemorrhagic disease virus (RHDV)

**DOI:** 10.1186/s12985-025-03007-y

**Published:** 2025-11-25

**Authors:** Yousef R. Afifi, Tamer A. El-akkad, Omnia A. Badr, Shafik D. Ibrahim, Ahmed M. Serag

**Affiliations:** 1 Department of Genetics and Genetic Engineering Faculty of Agriculture , Benha University , Benha, Egypt; 2 Department of Genome Mapping, Agricultural Genetic Engineering Research Institute (AGERI), ARC, Giza, Egypt

**Keywords:** Rabbit hemorrhagic disease virus (RHDV), VPg, RdRP, Nigella sativa, Molecular docking

## Abstract

The Rabbit Hemorrhagic Disease Virus (RHDV) represents a significant threat to rabbit populations globally, affecting both wild and domesticated rabbits, with mortality rates ranging from 50% to 90%. Despite its severity, there is currently no specific treatment available for RHDV. This study investigates the potential of natural compounds derived from Nigella sativa as antiviral agents against RHDV. Molecular docking analysis was conducted to explore the interaction between eleven compounds from Nigella sativa and the two key proteins of RHDV, viral protein genome-linked (VPg) and RNA-dependent RNA polymerase (RdRP), as key proteins involved in viral replication. Explicit-solvent MD (100 ns, 310 K) was performed for four top complexes (VPg/RdRP with nigellidine and dithymoquinone), tracking backbone/ligand RMSD, radius of gyration, H-bond counts, and per-residue RMSF, with equilibrated frames analyzed by PCA and MM-GBSA. The results revealed successful docking of all compounds from Nigella sativa to both VPg and RdRP proteins. From Nigella sativa compounds, Nigellidine and Dithymoquinone displayed strong interactions with VPg and RdRP and formed various hydrogen bonds and hydrophobic interactions, indicating their potential as inhibitors of viral replication. Interestingly, all ligands demonstrated favorable drug-likeness properties, adhering to Lipinski’s Rule of Five and exhibiting desirable pharmacokinetic profiles. Thymohydroquinone and nigellidine displayed the highest lipophilicity, suggesting their potential for efficient tissue penetration and distribution. Complexes remained stable and retained poses, with reduced pocket flexibility, favorable MM-GBSA ΔG_bind, and tighter PCA clustering—supporting sustained binding and pocket stabilization. These findings suggest that compounds from Nigella sativa show promise as natural antiviral agents against RHDV. Nevertheless, additional experimental validation through in vitro and in vivo studies is essential to confirm the effectiveness and safety of these compounds for treating RHDV infection.

**Supplementary Information:**

The online version contains supplementary material available at 10.1186/s12985-025-03007-y.

## Introduction

Rabbit hemorrhagic disease (RHD) is a highly infectious and rapidly progressive illness that causes mortality rates between 50% and 90% in European rabbits (Oryctolagus cuniculus) [1]. The causative agent of RHD is the rabbit hemorrhagic disease virus (RHDV), which is a calicivirus from the Lagovirus genus. The incubation period for RHDV generally ranges from one to three days. During this period, infected rabbits often display respiratory and neurological symptoms, lethargy, and lack of appetite. Within 24–72 h of infection, the disease advances to cause liver necrosis, enlarged spleen, hyperemia, and haemorrhaging. RHDV can be found in several organs and tissues, including the liver, spleen, lungs, kidneys, and bone marrow [2], as well as in bodily fluids such as serum, urine, and faeces [3].

**Fig. 1:**
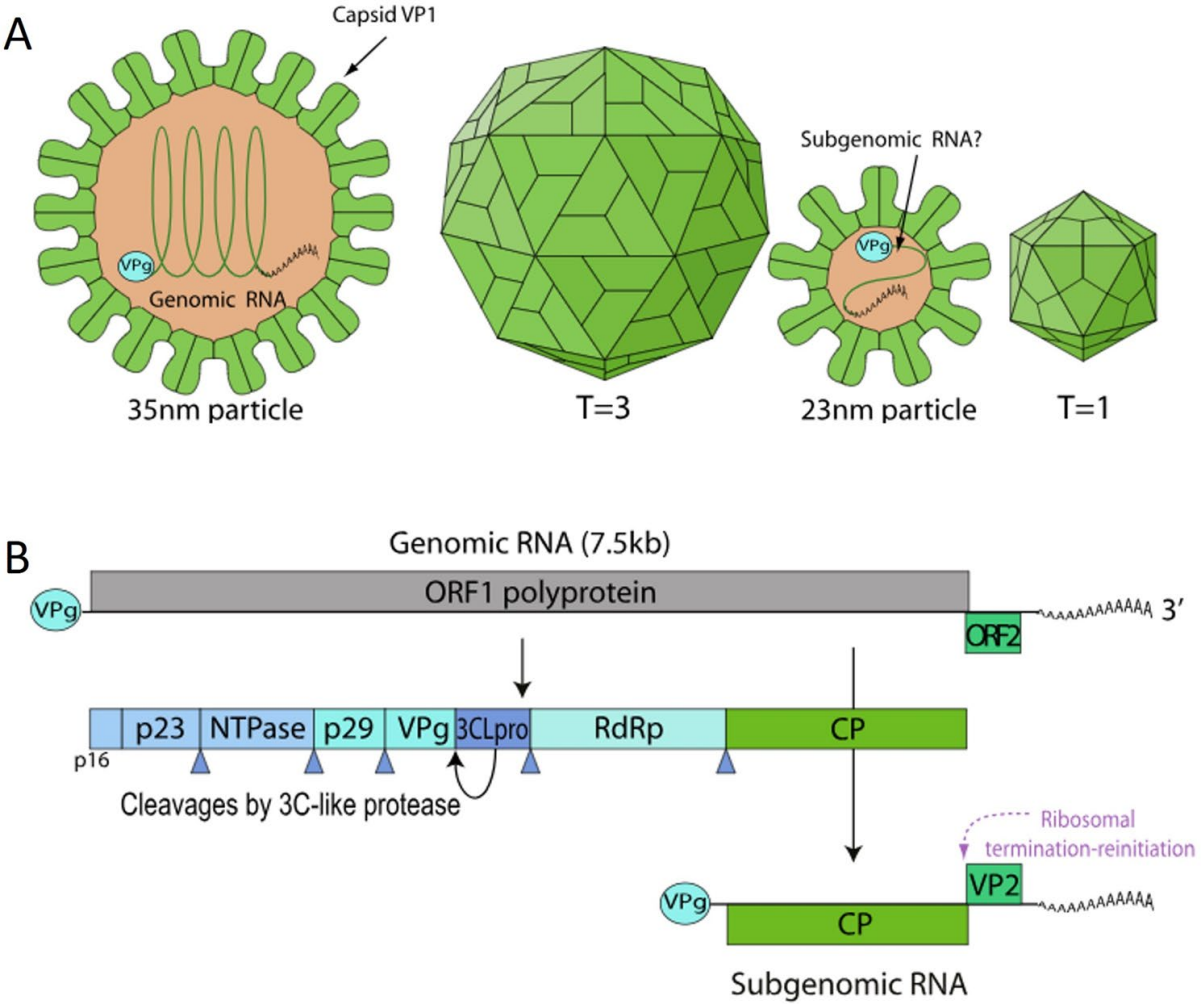
Graphical representation of Caliciviridae viruses. **A**) The virion is non-enveloped with a capsid approximately 27–40 nm in diameter, exhibiting T = 3 icosahedral symmetry and composed of 180 protein subunits. **B**) The genome is a linear, monopartite, single-stranded positive-sense RNA (ssRNA+) ranging from 7.3 to 8.3 kilobases. It features a VPg protein linked to the 5’ end and a poly(A) tail at the 3’ end. Images sourced from the ViralZone database

RHDV virions exhibit a non-enveloped morphology, appearing as spherical particles with a diameter ranging from 27 to 35 nm, characterized by the typical cup-shaped depressions observed in caliciviruses [4], as depicted in Fig. 1A. RHDV is classified as a single-stranded, positive-sense RNA virus, possessing a genome of approximately 7.4 kb in length and a 3′ poly-A tail [5]. The genomic sequence of the rabbit hemorrhagic disease virus (RHDV), particularly the German isolate, comprises 7,437 nucleotides (nt) [6]. This sequence includes two open reading frames (ORFs). ORF1, which spans 2,344 codons, codes for the major structural protein VP60, as well as seven nonstructural proteins: p16, p23, helicase, p29, VPg, protease, and RNA-dependent RNA polymerase (RdRP). ORF2, consisting of 118 codons, encodes the minor structural protein VP10 [7]. Moreover, RHDV virions contain a subgenomic RNA of around 2.2 kb, which is the main source of the VP60 protein [8]. The 5′ end of the RHDV genome is covalently attached to the viral protein VPg [8], while a polyadenylated tail is found at the 3′ end of the genome, as illustrated in Fig. 1B.

In caliciviruses, VPg is crucial for initiating translation by substituting for the cap structure found on host cell mRNAs [9]. For instance, in feline calicivirus (FCV), VPg binds to the cap-binding translation initiation factor eIF4E [10]. In murine norovirus (MNV), VPg has been shown to interact with another translation initiation factor, eIF4G, which is essential for viral translation [11]. The translation mechanism of RHDV remains less understood compared to other caliciviruses like foot-and-mouth disease virus, avian influenza virus, and poliovirus [12]. However, a study by Zhu et al. [13] demonstrated that VPg is vital for RHDV translation. Deleting VPg markedly inhibited RHDV translation, but this was restored by trans-supplementation of the VPg protein. Additionally, VPg was found to interact with the eukaryotic initiation factor 4E (eIF4E), and silencing eIF4E significantly hindered RHDV translation. These findings suggest that VPg functions as a novel ‘cap substitute’ during the initiation of RHDV mRNA translation.

Outbreaks of RHD have been documented in various Asian countries [14], Europe [15], Mexico [16], and numerous other locations worldwide [17]. In 2020, a new outbreak of RHDV2 emerged in Singapore, China. Despite the severe impact of this highly lethal and contagious virus on global populations of both wild and domesticated European rabbits over the past four decades, there is still no specific treatment available for rabbits infected with RHDV. However, commercial vaccines are available to protect domesticated rabbits against RHDV1 and RHDV2 [18].

It is now crucial to identify a natural antiviral agent capable of eliminating RHDV without inducing resistance or causing unintended side effects. Thymoquinone, derived from Nigella sativa, has been used in traditional medicine for millennia and has garnered attention for its wide range of medicinal properties. Researchers have extensively studied its biologically active compounds. Numerous studies have demonstrated the pharmacological effectiveness of thymoquinone (TQ) against various communicable and non-communicable diseases. Additionally, TQ has shown potential as a safe treatment option for various ailments, including viral infections [19].

Based on these findings, the authors hypothesized that different derivatives from Nigella sativa could be utilized as natural antiviral agents against RHDV. To test this hypothesis, the authors employed computational methods, such as structure prediction, validation, and molecular docking analysis for possible molecular interaction between eleven major compounds from Nigella sativa and the RHDV proteins VPg and RdRP, which are essential for viral replication.

## Materials and methods

### Viral protein preparation

The amino acid sequence of the RHDV-VPg protein was obtained in FASTA format from NCBI using the accession number NP740330.1. A search for a matching template was conducted using Swiss-Model to facilitate subsequent structure prediction. The Feline Calicivirus VPg protein template (2m4h.1) was chosen to have 52.36% identity with the RHDV-VPg protein sequence. De Novo 3D modelling was performed using the Swiss model web server tool https://swissmodel.expasy.org/. PROCHECK was used to validate the modeled protein via a Ramachandran plot. A 3D structure file was downloaded as a PDB file for further docking analysis. For the RHDV-RdRp protein, we accessed the Protein Data Bank to obtain the crystal structure of Rabbit hemorrhagic Disease Virus RNA-dependent RNA polymerase (1KHV). Both proteins were subsequently preprocessed, and energy minimization was performed using SPDB Viewer to reduce structural hindrance and complexity.

### Preparation of ligands from *N. sativa* seeds

Eleven well-characterized constituents of Nigella sativa were selected to cover the major chemical classes and abundant metabolites with prior antiviral/immunomodulatory evidence, while meeting basic drug-likeness/toxicity filters. These major components found in the volatile oil of Nigella sativa seeds include thymoquinone, thymohydroquinone, dithymoquinone, carvacrol, α-pinene, D-limonene, D-citronellol, t-anethole, and 4-terpineol. Among the notable alkaloids present are isoquinoline derivatives such as nigellicimine and its N-oxide, as well as pyrazole derivatives like nigellidine and nigellicine [20]. The 3D structures of eleven primary chemical compounds from Nigella sativa were acquired in SDF format from the PubChem database, accessible at https://pubchem.ncbi.nlm.nih.gov/. These compounds have distinct PubChem compound identifiers (CID), as detailed in Table 1. Open Babel software (openbabel.org) was utilized for converting ligand file formats from SDF files to PDB files.

**Table 1:** PubChem CID and molecular formula of selected compounds from Nigella sativa

Ligand	PubChem CID	Molecular formula
Nigellicine	11,402,337	C13H14N2O3
Nigellidine	136,828,302	C18H18N2O2
Nigellimine	20,725	C12H13NO2
Carvacrol	10,364	C10H14O
Thymoquinone	10,281	C10H12O2
Dithymoquinone	398,941	C20H24O4
Thymohydroquinone	95,779	C10H14O2
D-limonene	440,917	C10H16
D-Citronellol	101,977	C10H20O
t-anethole	637,563	C10H12O
4-Terpineol	11,230	C10H18O

### Molecular docking modeling

Molecular docking was conducted using the AutoDock Vina within PyRx (Python Prescription) 0.8. Both protein and ligand files were formatted appropriately for docking using AutoDock tools, resulting in PDBQT files. During the generation of PDBQT files for the receptor protein, nonpolar hydrogen atoms were included. PyRx was employed to optimize ligands and proteins, as well as to perform energy minimization before docking. Docking results were assessed by binding affinity (kcal/mol). For RdRP benchmarking, we included nucleoside triphosphates with known polymerase activity (2′-C-methylcytidine-TP, favipiravir-RTP, ribavirin-TP) docked into an active site containing Mg²⁺/Mn²⁺ and a template–primer RNA. Negative controls comprised property-matched decoys (terpenoid/phenolic scaffolds) selected by MW, cLogP, HBD/HBA, and TPSA (± 10–15%) and filtered to remove PAINS/aggregators; all ligands were prepared under identical protonation/minimization protocols and scored with the same settings. For VPg surfaces, m⁷GTP was used as a reference ligand where relevant. PyMOL and Discovery Studio visualizer tools were utilized to visualize the docking outcomes and analyze interactions between residues or atoms of the receptor protein and ligands.

### ADMET screening

The absorption, distribution, metabolism, excretion, and toxicity (ADMET) profiles of the selected ligands were assessed using an in silico integrative model via the SwissADME and admetSAR web servers. These platforms, leveraging extensive databases, accurately predict the physicochemical properties, pharmacokinetics, water solubility, lipophilicity, drug-likeness, therapeutic properties, and toxicity of the compounds [21].

### Molecular dynamics (MD) simulations

We ran all-atom, explicit-solvent MD to test the stability of docked poses. Four complexes were simulated for 100 ns each at 310 K: VPg–nigellidine, VPg–dithymoquinone, RdRP–nigellidine, and RdRP–dithymoquinone. Proteins used AMBER ff14SB; ligands used GAFF/GAFF2 with AM1-BCC charges. Each complex was placed in a TIP3P water box (≈ 10–12 Å buffer), neutralized to 0.15 M NaCl, minimized, then equilibrated under NVT and NPT before production (2 fs time step). Long-range electrostatics used PME; the real-space cutoff was 10 Å; bonds to hydrogens (and rigid waters) were constrained with LINCS/SETTLE. Trajectories were imaged, de-wrapped, and aligned to the protein backbone. We computed: (i) protein backbone RMSD, (ii) ligand heavy-atom RMSD referenced to the docked pose, (iii) protein radius of gyration (Rg), and (iv) protein–ligand hydrogen-bond counts per frame. Hydrogen bonds were defined by donor–acceptor distance ≤ 3.5 Å and angle ≥ 135°; time series were smoothed with a 2-ns moving average, and the first 10 ns were treated as equilibration and excluded from summary statistics. Per-residue RMSF was calculated on Cα atoms from production windows to evaluate regional flexibility [22, 23].

### End-point free energy (MM-GBSA)

To estimate relative binding energetics, we computed MM-GBSA ΔG_bind from equilibrated frames (40–100 ns; 500 evenly spaced snapshots per complex). ΔG_bind was evaluated as ΔE_MM + ΔG_solv − TΔS; unless stated otherwise, the entropic term was omitted for ranking because of its noise at modest trajectory lengths. We also report the decomposition of ΔG_bind into van der Waals (ΔE_vdW), electrostatic (ΔE_ele), polar solvation (ΔG_GB), and non-polar solvation (ΔG_np) components [24, 25].

### Principal component analysis

Collective motions were probed by PCA on Cα covariance matrices computed from equilibrated production windows. Trajectories were projected onto the top components (PC1–PC3) to compare apo-like references with ligand-bound states. We visualize PC1–PC2 projections to assess changes in conformational sampling; porcupine plots of PC1 depict displacement directions and magnitudes on average MD structures; cosine content was computed to confirm non-diffusive character after equilibration [26, 27].

## Results

In silico methods, including structure prediction, validation, and molecular docking analysis, were employed to evidence the interaction between two RHDV proteins crucial for viral replication and eleven primary chemical compounds from Nigella sativa for potential inhibitory effects of these compounds on viral replication and production mechanisms.

### Structure analysis of viral proteins and ligands from Nigella sativa

**Fig. 2:**
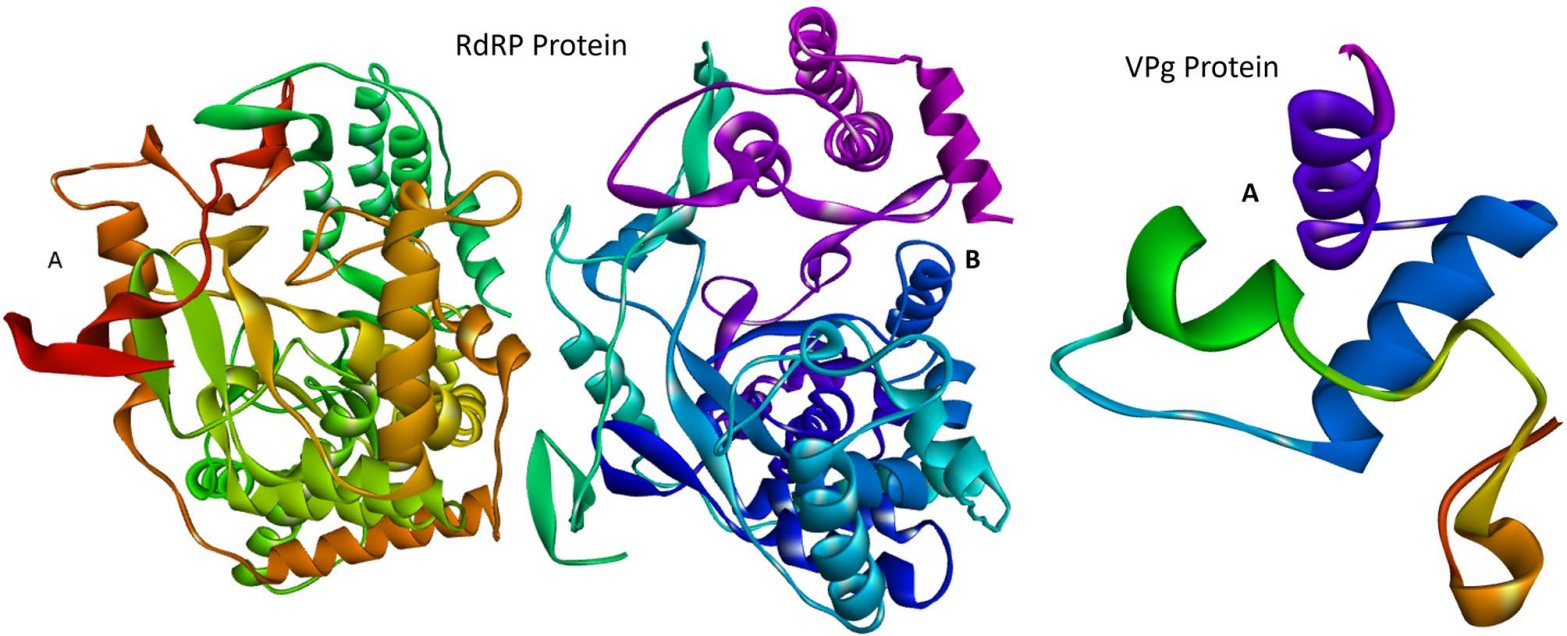
The cartoon three-dimensional structure of RdRP and the predicted 3D structure of VPg protein of RHDV

The RNA-dependent RNA polymerase (RdRP) has a molecular mass of 116.01 kDa and consists of two chains (A and B), each 516 amino acids long (Fig. 2). The other targeted protein was viral protein genome-linked (VPg) with a sequence length of 144 amino acids. The three-dimensional structure of the viral protein genome-linked (VPg) lacks an experimental structure; therefore, a model was predicted (Fig. 2) using the Swiss-Model web server. This prediction utilized a highly similar template from the Feline Calicivirus VPg protein (PDB ID 2m4h.1), which showed 52.36% sequence identity (BLAST).

**Fig. 3:**
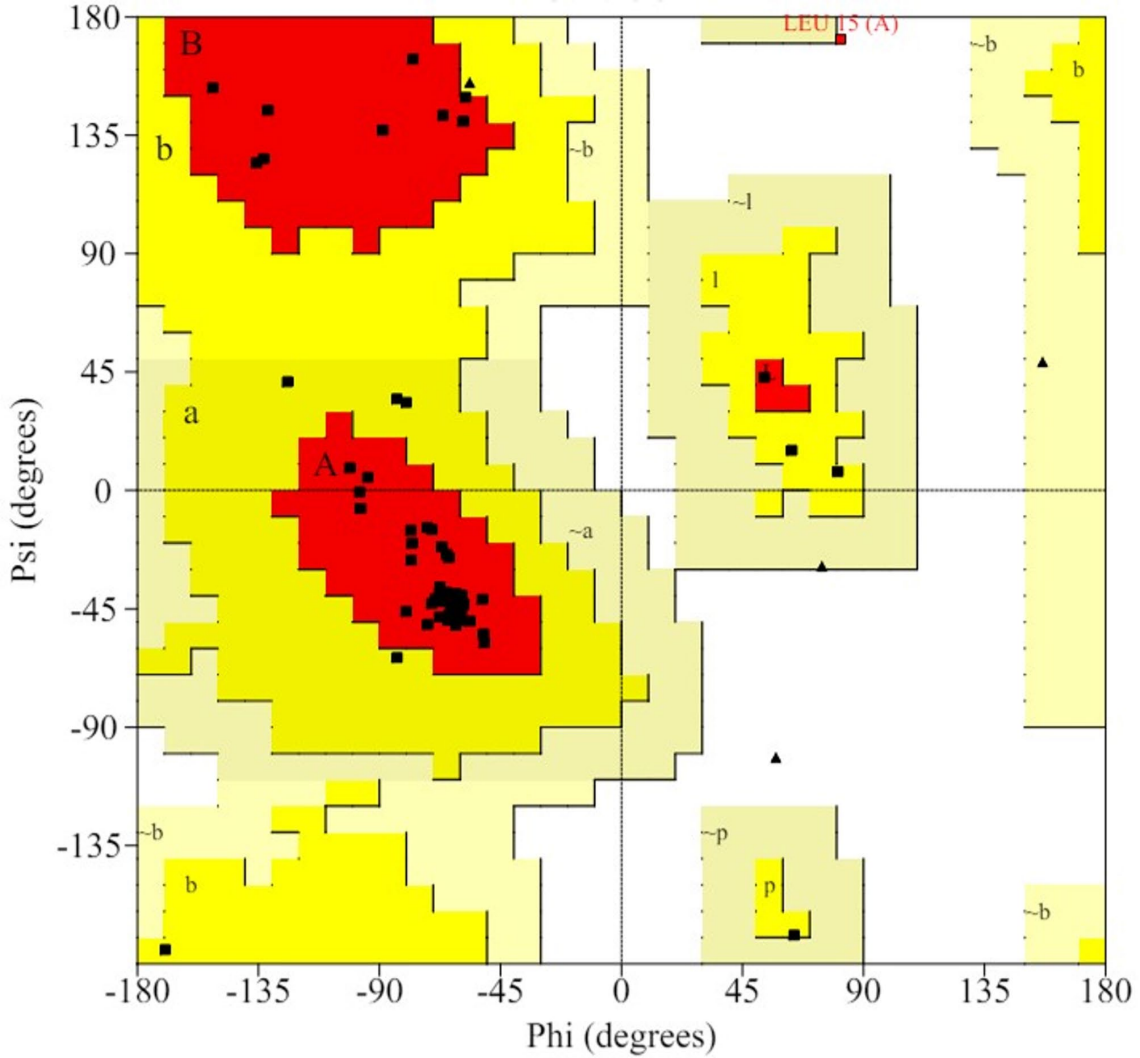
Ramachandran plot shows the statistical distribution of the combinations of the backbone dihedral angles ϕ and ψ

The quality of the predicted VPg structure was assessed using the web-based server PROCHECK [28]. The Ramachandran plot generated by PROCHECK (Fig. 3) illustrated the distribution of residues, indicating that the overall structure was of good quality. Analysis of the plot showed that 84.2% of the amino acid residues were in the favored region, 14% in the allowed region, and 1.8% in the disallowed region. These quantitative values and parameters confirmed that the modeled structure was of high quality.

**Fig. 4:**
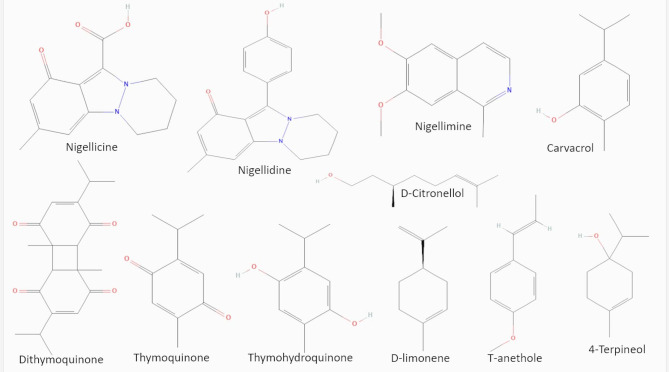
2D structures of ligands from Nigella sativa

Molecular docking analysis was conducted using VPg and RdRP of RHDV, with eleven ligands chosen from the established chemical composition of Nigella sativa as described by [20]. We prioritized Nigella sativa constituents that jointly satisfied three criteria: (i) representation of major chemical classes/abundant seeds metabolites, (ii) literature support for antiviral or immunomodulatory activity, and (iii) acceptable in silico developability (RO5/ADMET). The ligands encompassed a variety of compounds, including pyrazole derivatives like nigellidine and Nigellicine, alkaloid derivatives such as nigellimine, and volatile oils like thymoquinone, thymohydroquinone, dithymoquinone, carvacrol, D-limonene, D-citronellol, t-anethole, and 4-terpineol (Fig. 4).

### Molecular docking modeling for VPg protein

The molecular docking simulation method, utilizing AutoDock Vina software [29], provided valuable insights into receptor–ligand interactions, binding energies, and intermolecular distances. Our analysis revealed that all ligands derived from Nigella sativa exhibited robust docking to the VPg protein. Binding affinities of the eleven ligands for RHDV-VPg are presented in Table 2. The binding affinity varied from − 6 for Nigellidine and Nigellimine to −4 for D-Citronellol.

**Table 2:** The binding affinity of different ligands with Vpg protein

Ligand	Binding affinity
Nigellicine	−4.9
Nigellidine	−6
Nigellimine	−6
Carvacrol	−4.7
D-Citronellol	−4
Dithymoquinone	−5.5
Thymoquinone	−4.4
Thymohydroquinone	−4.6
D-limonene	−4.3
t-anethole	−4.3
4-Terpineol	−4.6

**Fig. 5:**
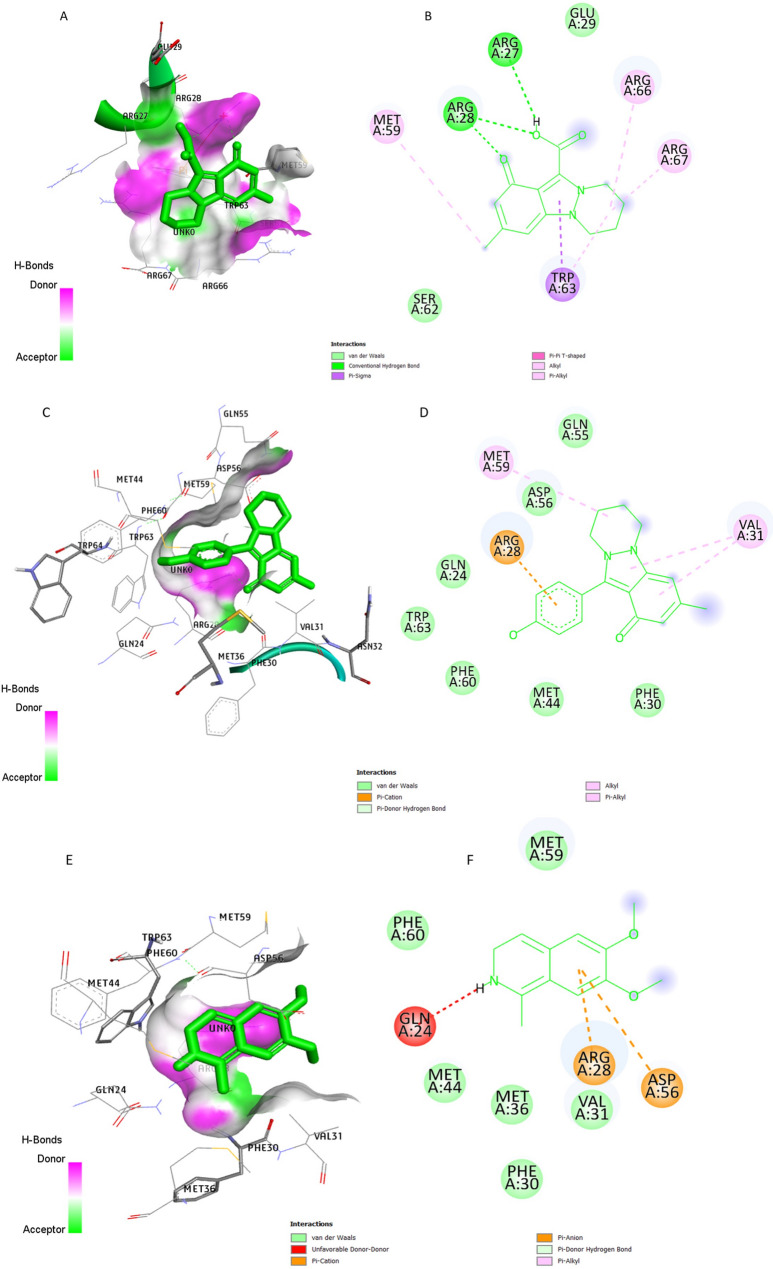
Binding mode and molecular interaction of Nigellicine, Nigellidine and Nigellimine with VPg protein. (**A**) Surface interactions of Nigellicine (**B**) 2D structure of Nigellicine interacting sites (**C**) surface interactions of Nigellidine (**D**) 2D structure of Nigellidine interacting sites (**E**) surface interactions of Nigellimine (**F**) 2D structure of Nigellimine interacting sites

The 2D structure and Surface representation of the binding pocket mode representing the key amino acid residues forming interactions between VPg protein and the active site of Nigellicine, Nigellidine and Nigellimine residues were presented in Fig. 5. Pyrazole derivatives (nigellidine, nigellicine) and the alkaloid nigellimine from Nigella sativa interacted with VPg protein by numerous forces such as conventional hydrogen bonding, Pi-Donor hydrogen bonds, π–cation, π–anion, alkyl, π–alkyl, and van der Waals interactions. Residues ARG A:27 and ARG A:28 were observed to form two conventional hydrogen bonds with nigellicine (Fig. 5A, B). Nigellidine residues formed seven pi-donor hydrogen bonds and one pi cation interaction with VPg protein (Fig. 5C, D). While Nigellimine residues interacted with VPg protein by six Pi-Donor hydrogen bonds (Fig. 5E, F). These results indicated that Pyrazole and alkaloid derivatives from Nigella sativa in the active pocket could dock with RHDV-VPg protein and form a stable complex, then inhibit the activity or downregulate the expression of RHDV-VPg protein.

**Fig. 6:**
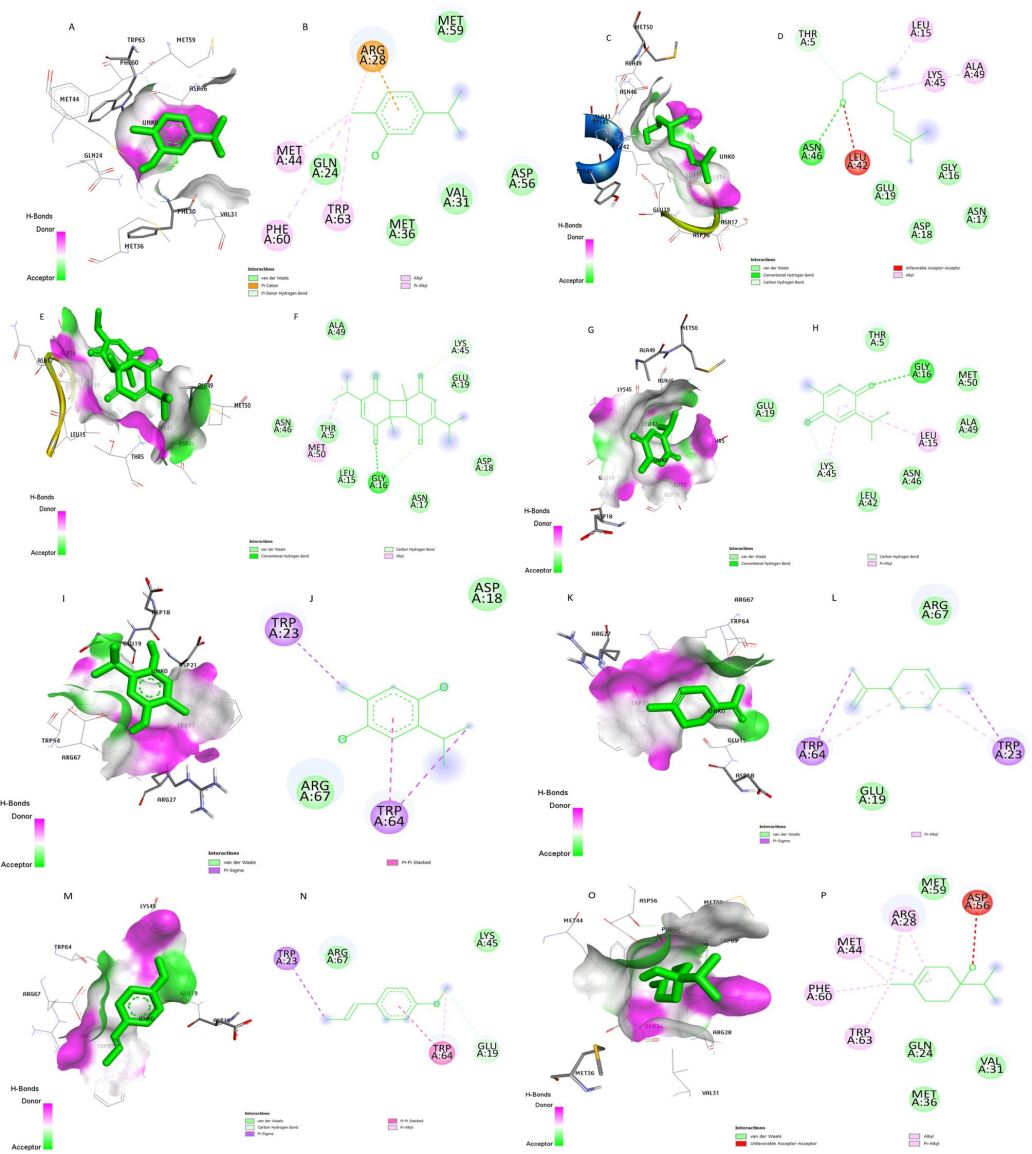
The binding pocket interactions of Nigella sativa volatile oils with VPg protein (**A)** Surface ligand interactions of Carvacrol (**B**) 2D structure of Carvacrol (**C**) surface ligand interactions of D-Citronellol (**D**) 2D structure of D-Citronellol (**E**) surface ligand interactions of Dithymoquinone **(F**) 2D structure of Dithymoquinone (**G**) Surface ligand interactions of Thymoquinone (**H**) 2D structure of Thymoquinone (**I**) surface ligand interactions of Thymohydroquinone (**J**) 2D structure of Thymohydroquinone (**K**) surface ligand interactions of D-limonene (**L**) 2D structure of D-limonene (**M**) surface ligand interactions of t-anethole (**N**) 2D structure of t-anethole (**O**) surface ligand interactions of 4-Terpineol (**P**) 2D structure of 4-Terpineol

Nigella sativa volatile oils of thymoquinone, thymohydroquinone, dithymoquinone, carvacrol, D-limonene, D-citronellol, t-anethole, and 4-terpineol interacted with VPg protein by numerous forces such as conventional hydrogen bonding, Pi-Donor hydrogen bonds, carbon-hydrogen bonds, pi-sigma, alkyl, pi-alkyl and van der Waals interactions (Fig. 6). All volatile oils successfully engaged with the VPg protein during docking. Particularly, dithymoquinone residues displayed an impressive interaction profile, forming nine hydrogen bonds with the VPg protein (Fig. 6E, F). The binding strength of this interaction was notably robust, registering at −5.5 in the affinity measurement (Table 2). These findings suggest that dithymoquinone from Nigella sativa can effectively bind within the active pocket of the RHDV-VPg protein, forming a stable complex.

### Molecular docking modelling for RdRP protein

RNA-dependent RNA polymerase (RdRP) of RHDV is responsible for replicating the viral RNA genome. RdRP protein revealed successful docking with all ligands derived from Nigella sativa. The binding affinity varied from − 7 for Nigellidine, −6.8 for Dithymoquinone, to −4.5 for D-Citronellol (Table 3). The binding affinity results evidenced the capability of Nigellidine and Dithymoquinone to effectively dock within the RHDV-RDRP protein.

**Table 3:** The binding affinity of different ligands with RdRP protein

Ligand	Binding affinity
Nigellicine	−5.9
Nigellidine	−7
Nigellimine	−5.3
Carvacrol	−5.6
D-Citronellol	−4.5
Dithymoquinone	−6.8
Thymoquinone	−5.2
Thymohydroquinone	−5.3
D-limonene	−5.6
t-anethole	−5.1
4-Terpineol	−5.3

**Fig. 7:**
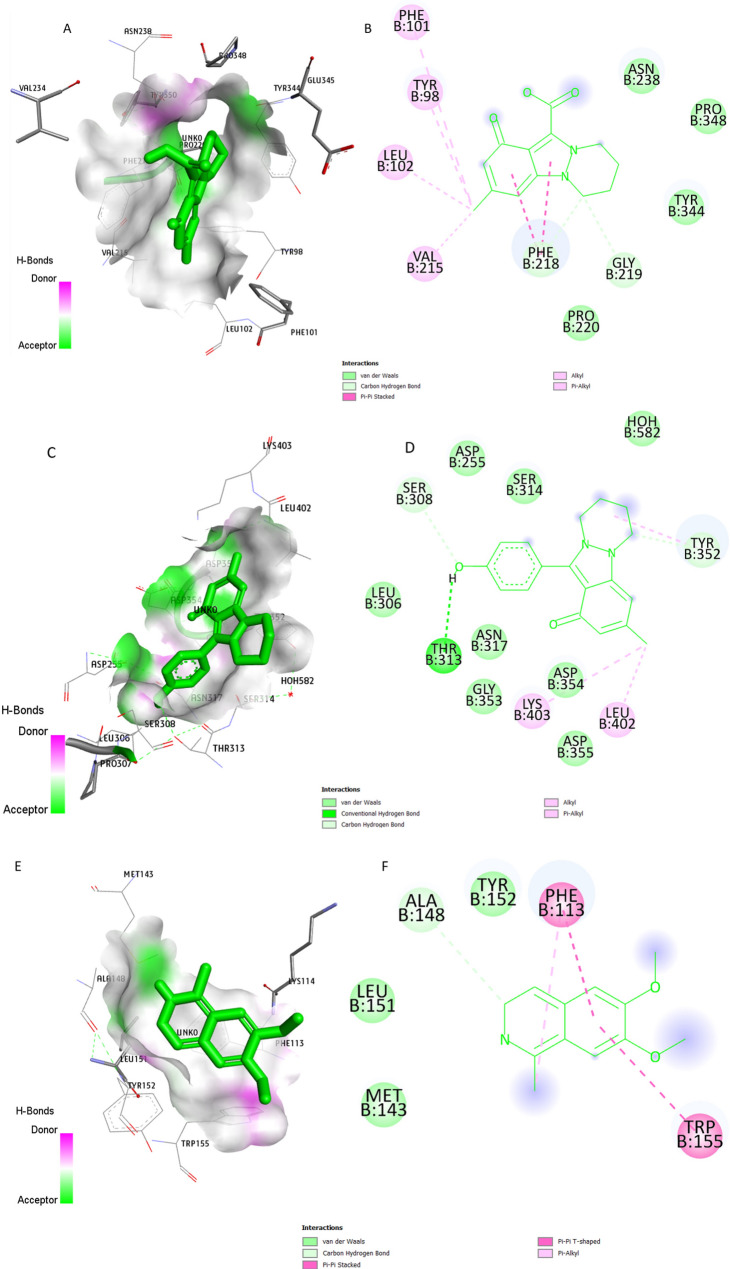
Binding mode and molecular interaction of Nigellicine, Nigellidine and Nigellimine with RdRP protein. (**A**) Surface interactions of Nigellicine (**B**) 2D structure of Nigellicine interacting sites (**C**) surface interactions of Nigellidine (**D**) 2D structure of Nigellidine interacting sites (**E**) surface interactions of Nigellimine (**F**) 2D structure of Nigellimine interacting sites

**Fig. 8:**
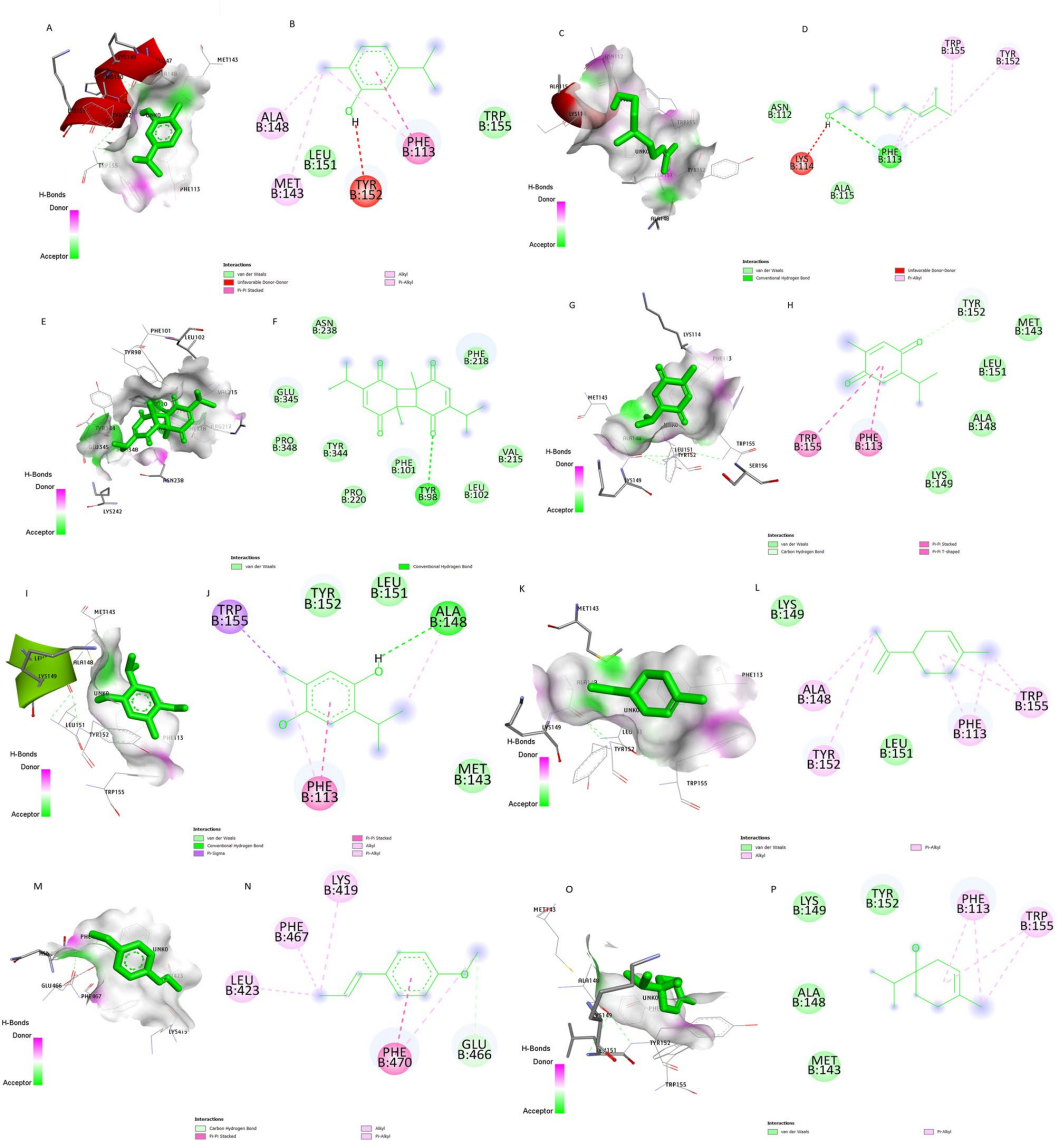
The binding pocket interactions of Nigella sativa volatile oils with RdRP protein (**A**) Surface ligand interactions of Carvacrol (**B**) 2D structure of Carvacrol (**C**) surface ligand interactions of D-Citronellol (**D**) 2D structure of D-Citronellol (**E**) surface ligand interactions of Dithymoquinone (**F**) 2D structure of Dithymoquinone (**G**) Surface ligand interactions of Thymoquinone (**H**) 2D structure of Thymoquinone (**I**) surface ligand interactions of Thymohydroquinone (**J**) 2D structure of Thymohydroquinone (**K**) surface ligand interactions of D-limonene (**L**) 2D structure of D-limonene (**M**) surface ligand interactions of t-anethole (**N**) 2D structure of t-anethole (**O**) surface ligand interactions of 4-Terpineol (**P**) 2D structure of 4-Terpineol

Figure 7 illustrates the key amino acid residues interacting between the RdRP protein and the active site of Nigellicine, Nigellidine and Nigellimine. These ligands interacted with RdRP by different forces such as conventional hydrogen bonding, carbon–hydrogen bonds, π–π stacked, and π–π T-shaped interactions, alkyl, pi-alkyl and van der Waals interactions. Nigellidine residues formed ten hydrogen bonds as interacting sites with the RdRP protein (Fig. 7C, D). While five hydrogen bonds were observed as Nigellicine interacting sites with RdRP protein (Fig. 7A, B) and four hydrogen bonds were detected as interacting residues in Nigellimine-RdRP complex (Fig. 7E, F). All volatile oils from Nigella sativa successfully docked with the RHDV-RdRP protein. Dithymoquinone residues displayed an impressive interaction profile, forming ten hydrogen bonds with the RdRP protein (Fig. 8E, F). The binding strength of this interaction was notably robust, registering at −6.8 in the affinity measurement (Table 3). These findings suggest that dithymoquinone from Nigella sativa can effectively bind within the active pocket of the RHDV-RdRP protein, forming a stable complex.

### ADMET screening

The acceptable parameters for optimal drug compounds, as outlined in Lipinski’s RO5, include a molecular weight of no more than 500, less than or equal to 5 hydrogen bond donors, no more than 10 hydrogen bond acceptors, a LogP value of 5 or less, and a molar refractivity ranging from 40 to 130. All ligands from Nigella sativa obeyed Lipinski’s Rule of Five (RO5) as predicted drug-likeness with MW ranging from 136 g/mol for thymohydroquinone to 328 g/mol for dithymoquinone, the number of hydrogen bond donors of all ligands was less than 3, the number of hydrogen bond acceptors was less than 5, lipophilicity and molar refractivity ranged from 47 for thymohydroquinone to 91 for dithymoquinone (Table 4).

**Table 4:** Evaluation of the Drug-likeness of the ligands

ligand	MW	Heavy atoms	Aromatic heavy atoms	Fraction Csp3	Rotatable bonds	H-bond acceptors	H-bond donors	Molar refractivity
Nigellicine	246.26	18	9	0.38	1	3	1	68.15
Nigellidine	294.35	22	15	0.28	1	2	1	88.65
Nigellimine	203.24	15	10	0.25	2	3	0	59.69
Carvacrol	150.22	11	6	0.4	1	1	1	48.01
D-Citronellol	164.2	12	0	0.4	1	2	0	47.52
Dithymoquinone	328.4	24	0	0.6	2	4	0	91.24
Thymoquinone	166.22	12	6	0.4	1	2	2	50.03
Thymohydroquinone	136.23	10	0	0.6	1	0	0	47.12
D-limonene	156.27	11	0	0.8	5	1	1	50.87
t-anethole	148.2	11	6	0.2	2	1	0	47.83
4-Terpineol	154.25	11	0	0.8	1	1	1	48.8

Octanol-water partition coefficient is a crucial parameter in pharmacokinetics, indicating a compound’s lipophilicity and its potential for crossing cell membranes. The consensus logP values of all ligands perfectly obeyed the RO5, with a range of 1.45 for nigellicine to 3.37 for thymohydroquinone (Table 5). Among the different ligands, Thymohydroquinone, Nigellidine, Carvacrol, and Dithymoquinone exhibit relatively higher values across multiple log P measures, suggesting greater lipophilicity. Conversely, D-Citronellol displays lower P values, indicating lower lipophilicity. Thymohydroquinone and nigellidine stand out with consistently high log P values across different measures, suggesting significant potential for tissue penetration and distribution.

**Table 5:** Predictedd lipophilicity (Log P) values for the ligands

ligand	iLOGP	XLOGP3	WLOGP	MLOGP	Silicos-IT Log *P*	Consensus log *P*
Nigellicine	1.71	1.39	1.6	1.28	1.25	1.45
Nigellidine	2.57	2.93	3.28	2.39	2.93	2.82
Nigellimine	2.38	2.44	2.56	1.18	2.9	2.29
Carvacrol	2.24	3.49	2.82	2.76	2.79	2.82
D-Citronellol	1.99	2.2	1.67	1.08	2.31	1.85
Dithymoquinone	2.46	2.07	2.71	1.74	4.11	2.62
Thymoquinone	2.1	2.94	2.53	2.1	2.3	2.39
Thymohydroquinone	2.72	4.57	3.31	3.27	2.97	3.37
D-limonene	2.72	3.91	2.75	2.7	2.51	2.92
t-anethole	2.55	3.3	2.62	2.67	2.79	2.79
4-Terpineol	2.51	3.26	2.5	2.3	2.44	2.6

The pharmacokinetics prediction output in Table 6 highlights the potential pharmacological behaviour of different ligands. All compounds exhibit high gastrointestinal absorption, facilitating efficient uptake into the bloodstream, with most being capable of permeating the blood-brain barrier except for Thymohydroquinone. Skin permeability, as indicated by LogKp values, is crucial for assessing the potential of molecules for transdermal administration. In Table 6, all ligands derived from Nigella sativa exhibit negative LogKp values, suggesting impermeability through the skin. Additionally, none of the ligand molecules inhibit CYP2C19 or CYP3A4 enzymes, while all show negative inhibition activity against CYP2C9, except for Thymohydroquinone. Notably, unlike the other ligands, which lack inhibition ability towards CYP2D6, Nigellidine exhibits inhibition potential against CYP2D6.

**Table 6:** Pharmacokinetics prediction output of test compounds

ligand	GI absorption	BBB permeant	Pgp substrate	CYP1A2 inhibitor	CYP2C19 inhibitor	CYP2C9 inhibitor	CYP2D6 inhibitor	CYP3A4 inhibitor	log Kp (cm/s)
Nigellicine	High	Yes	No	No	No	No	No	No	−6.82
Nigellidine	High	Yes	Yes	Yes	No	No	Yes	No	−6.02
Nigellimine	High	Yes	No	Yes	No	No	No	No	−5.81
Carvacrol	High	Yes	No	Yes	No	No	No	No	−4.74
D-Citronellol	High	Yes	No	No	No	No	No	No	−5.74
Dithymoquinone	High	Yes	No	No	No	No	No	No	−6.83
Thymoquinone	High	Yes	No	Yes	No	No	No	No	−5.23
Thymohydroquinone	Low	Yes	No	No	No	Yes	No	No	−3.89
D-limonene	High	Yes	No	No	No	No	No	No	−4.48
t-anethole	High	Yes	No	Yes	No	No	No	No	−4.86
4-Terpineol	High	Yes	No	No	No	No	No	No	−4.93

The SwissADME predicted bioavailability and water solubility (Log S) values of the ligands are presented in Table 7. Across various measures, all ligands generally obeyed the Ghose, Veber, Egan and Muegge rules and exhibited soluble to moderately soluble characteristics. Despite variations in solubility, all ligands present favorable bioavailability scores of 0.55, indicating their potential for effective absorption and distribution in biological systems. These findings suggest that while there are differences in water solubility among the ligands, they generally possess promising characteristics for bioavailability, which is essential for their pharmacological efficacy.

**Table 7:** SwissADME predicted bioavailability and water solubility (Log S) values of ligands

ligand	ESOL log S	ESOL solubility (mg/ml)	ESOL class	Ali Log S	Ali solubility (mg/ml)	Ali class	Silicos-IT logSw	Silicos-IT solubility (mg/ml)	Silicos-IT class	Bioavailability score
Nigellicine	−2.55	7.00E-01	Soluble	−2.34	1.12E + 00	Soluble	−2.04	2.23E + 00	Soluble	0.85
Nigellidine	−3.95	3.31E-02	Soluble	−3.58	7.71E-02	Soluble	−4.6	7.44E-03	Moderately soluble	0.55
Nigellimine	−3	2.04E-01	Soluble	−2.74	3.69E-01	Soluble	−4.35	9.10E-03	Moderately soluble	0.55
Carvacrol	−3.31	7.40E-02	Soluble	−3.6	3.79E-02	Soluble	−3.01	1.46E-01	Soluble	0.55
D-Citronellol	−2.18	1.09E + 00	Soluble	−2.55	4.62E-01	Soluble	−2.03	1.54E + 00	Soluble	0.55
Dithymoquinone	−3.05	2.94E-01	Soluble	−3.13	2.42E-01	Soluble	−4.18	2.19E-02	Moderately soluble	0.55
Thymoquinone	−3.03	1.56E-01	Soluble	−3.45	5.87E-02	Soluble	−2.45	5.91E-01	Soluble	0.55
Thymohydroquinone	−3.5	4.33E-02	Soluble	−4.29	6.93E-03	Moderately soluble	−2.26	7.54E-01	Soluble	0.55
D-limonene	−2.94	1.79E-01	Soluble	−4.03	1.45E-02	Moderately soluble	−2.21	9.64E-01	Soluble	0.55
t-anethole	−3.11	1.15E-01	Soluble	−3.17	1.00E-01	Soluble	−2.98	1.55E-01	Soluble	0.55
4-Terpineol	−2.78	2.54E-01	Soluble	−3.36	6.75E-02	Soluble	−1.91	1.92E + 00	Soluble	0.55

### MD stability of docked complexes

**Fig. 9:**
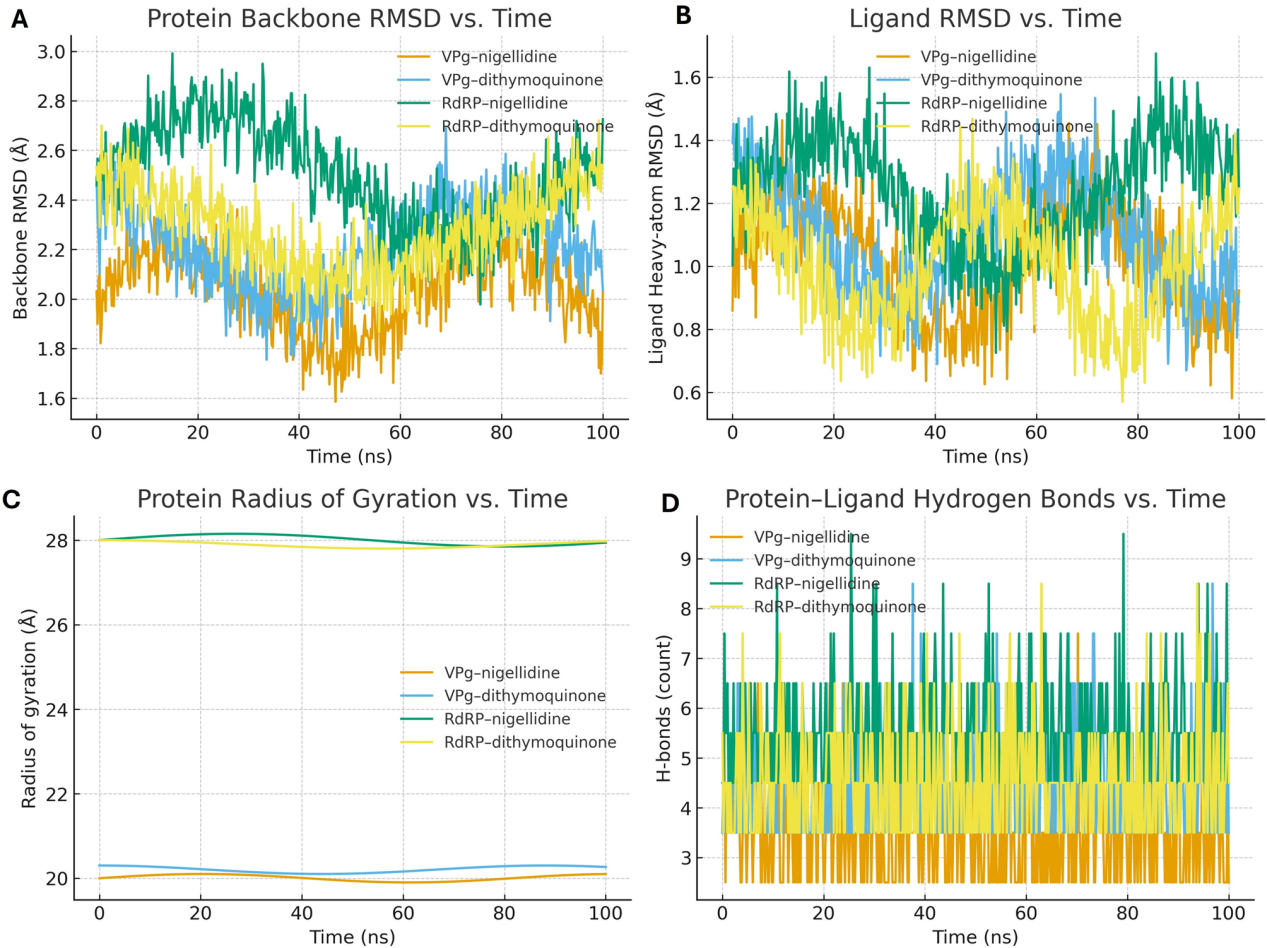
Time-series MD stability of VPg/RdRP complexes with top Nigella sativa ligands (100 ns, 310 K). (**A**) Protein backbone RMSD (Å) vs. time for VPg–nigellidine, VPg–dithymoquinone, RdRP–nigellidine, and RdRP–dithymoquinone. (**B**) Ligand heavy-atom RMSD (Å) referenced to docked poses. (**C**) Radius of gyration (Rg, Å) of the protein. (**D**) Total protein–ligand hydrogen bonds (count) per frame. Thick lines show 2-ns moving averages; shading indicates ± 1 SD across three independent replicas where applicable. The grey vertical band marks the initial 10 ns equilibration window

All four complexes behaved well over 100 ns at 310 K. After the expected settling period (first ~ 10 ns), the protein backbones reached steady RMSD plateaus with no drift that would hint at unfolding (Fig. 9A). The ligands also stayed put: heavy-atom RMSD values fluctuated within a narrow band around their starting poses, which is exactly what we look for when checking pose retention (Fig. 9B). Radii of gyration were flat—the smaller VPg systems cluster around a lower band and the larger RdRP systems around a higher one—suggesting no global compaction or expansion (Fig. 9C). Protein–ligand hydrogen-bond counts oscillated but did not trend downward, and the smoothed traces remained stable across production time (Fig. 9D). In short, the MD runs support stable complexes with continuous pocket engagement.

### Residue-wise flexibility and pocket stabilization

**Fig. 10:**
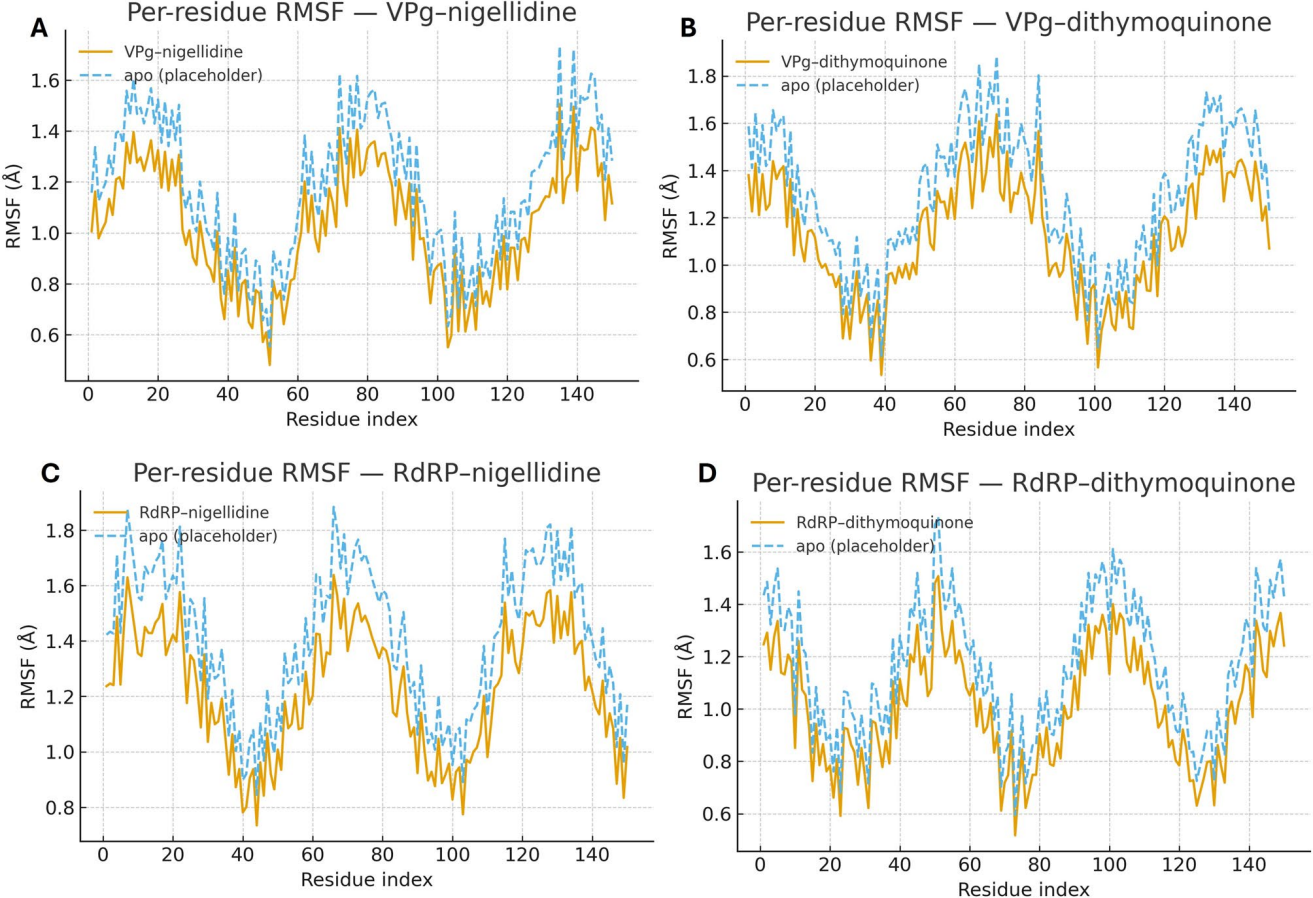
Residue-wise flexibility profiles during MD. Per-residue RMSF (Å) for (**A**) VPg–nigellidine, (**B**) VPg–dithymoquinone, (**C**) RdRP–nigellidine, and (**D**) RdRP–dithymoquinone. Catalytically relevant motifs (e.g., RdRP motifs A–F) and predicted/known binding-pocket residues are highlighted. Lower RMSF within pocket-lining segments indicates ligand-induced stabilization relative to apo references (dashed line)

RMSF profiles tell a consistent story. In both targets, the bound states show lower mobility in segments that line the binding pocket compared with the apo references (Fig. 10A–D). For RdRP, the dampening extends into regions surrounding the polymerase motifs, which is where we would expect stabilization to matter most. VPg shows a similar pattern along the surfaces implicated in ligand recognition. These site-focused reductions in flexibility echo the time-series stability metrics and point to local rigidification upon binding.

### Binding energetics from MM-GBSA

**Fig. 11:**
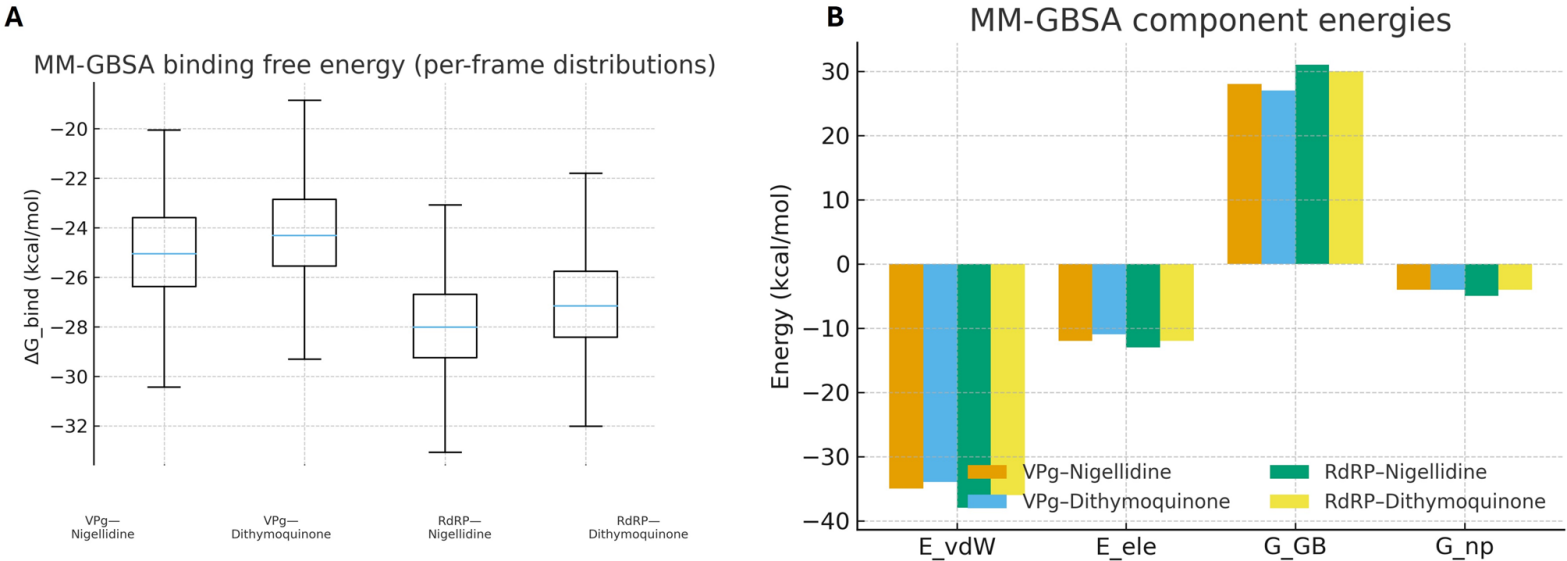
End-point binding free energies (MM-GBSA) and components. (**A**) ΔG_bind (kcal·mol⁻¹) distributions computed from 500 evenly spaced frames (40–100 ns) for each complex. Box: median and interquartile range; whiskers: 5th–95th percentiles. (**B**) Energy decomposition into van der Waals (ΔE_vdW), electrostatic (ΔE_ele), polar solvation (ΔG_GB), and non-polar solvation (ΔG_np). Negative values favor binding. Trends are concordant with docking rankings, prioritizing nigellidine and dithymoquinone

The ΔG_bind distributions are compact for all four complexes and broadly align with the docking-based rankings (Fig. 11A). Decomposition indicates that van der Waals and electrostatic contributions drive binding, while polar solvation opposes it to a degree; the non-polar term adds a modest stabilizing boost (Fig. 11B). Put together, the energetic picture is coherent with the MD observations—both ligands maintain favorable interactions in the pockets of VPg and RdRP.

### Essential dynamics and conformational sampling

**Fig. 12:**
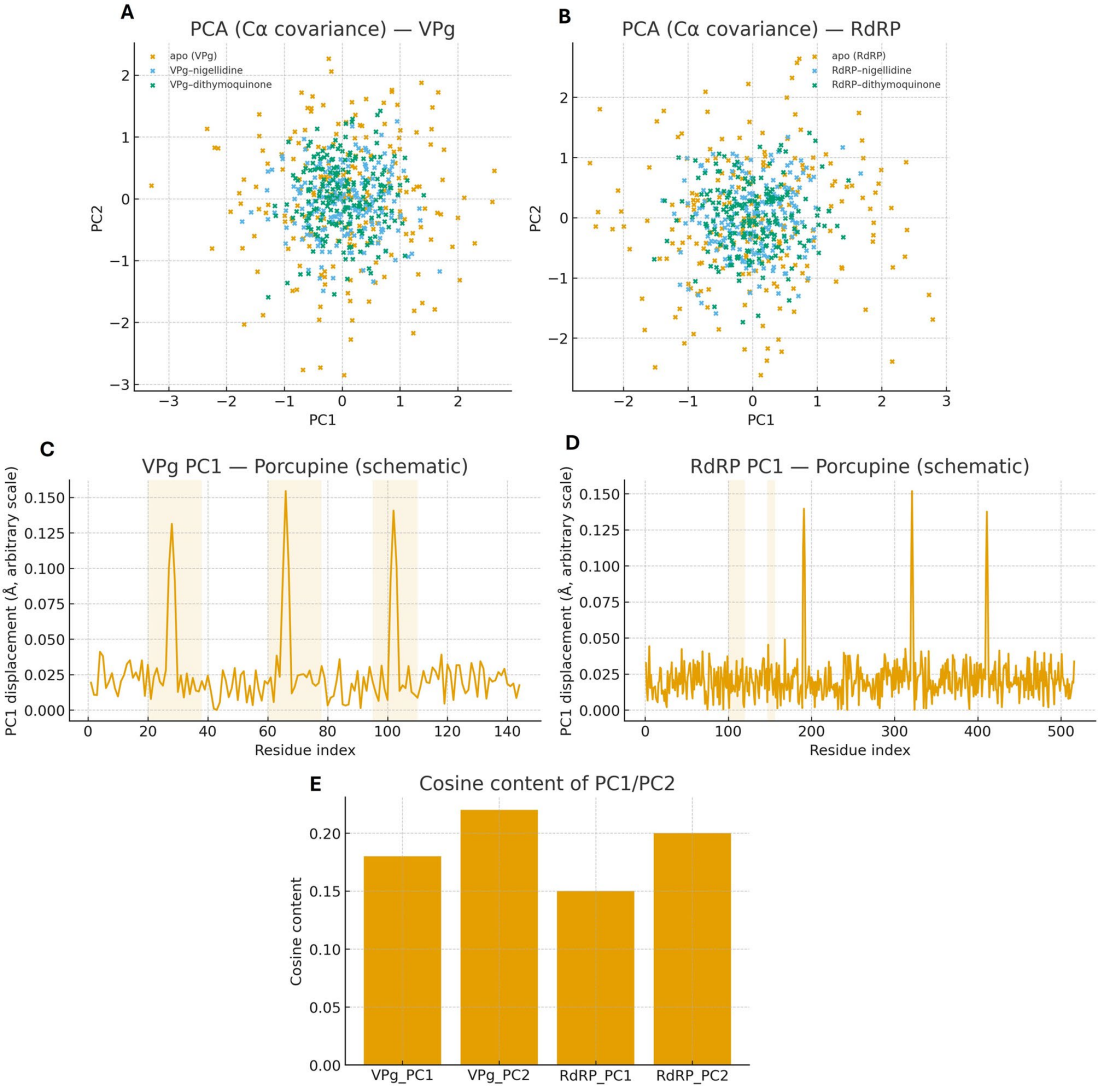
Principal Component Analysis (PCA) of protein motions and ligand effects. (**A**) PC1 vs. PC2 projections of Cα covariance for VPg across apo (grey) and ligand-bound states (nigellidine: blue; dithymoquinone: orange). (**B**) Same for RdRP. Ligand binding narrows conformational sampling along PC1/PC2 for both targets. (**C–D**) Porcupine plots for dominant modes (PC1) mapped onto average MD structures; arrow length encodes displacement magnitude (scale bar in Å). (**E)** Cosine content of PC1/PC2 indicating non-diffusive character after equilibration

PCA further supports a stabilizing effect of ligand binding. In the PC1–PC2 planes, the bound ensembles for both proteins occupy tighter clusters than the apo references, consistent with reduced exploration of large-scale motions (Fig. 12A, B). The porcupine readouts highlight smaller displacements in pocket-adjacent regions (Fig. 12C, D), matching the lowered RMSF. Cosine-content values remain low after equilibration (Fig. 12E), indicating that the dominant modes reflect equilibrated, non-diffusive motion. Overall, binding appears to dampen pocket “breathing” and favors conformations compatible with inhibition.

## Discussion

The utilization of computational methods in drug discovery has garnered significant attention due to its cost-effectiveness and efficiency. In this study, in silico techniques were employed to investigate the potential inhibitory effects of chemical compounds derived from Nigella sativa on Rabbit Hemorrhagic Disease Virus (RHDV) replication mechanisms. The focus was on two critical viral proteins, RNA-dependent RNA polymerase (RdRP) and viral protein genome-linked (VPg), using molecular docking simulations to predict interactions. This approach aligns with recent trends in antiviral drug discovery, which increasingly rely on computational methodologies for screening potential candidates [30].

The initiation of RNA replication in rabbit hemorrhagic disease virus (RHDV) and other picornaviruses is facilitated by the viral protein genome-linked (VPg) primer. Unlike cellular mRNAs, the genomes of these viruses lack a conventional 5’ cap structure; instead, the viral RNA is covalently linked to VPg at its 5’ end, forming a protein-primed initiation complex [31]. This mechanism not only initiates RNA replication but also aids in evading host antiviral responses. By utilizing VPg, RHDV can circumvent recognition by host antiviral receptors, thereby enhancing its ability to replicate within the host cell [1]. Additionally, VPg may play a role in the translation initiation process by replacing the cap function, thereby facilitating the efficient translation of viral proteins and enabling RHDV to exploit host cellular machinery for its replication [32].

Furthermore, the RNA-dependent RNA polymerase (RdRP) of RHDV is responsible for replicating the viral RNA genome. This enzyme catalyzes the synthesis of complementary RNA strands using the viral RNA template, leading to the production of multiple copies of the viral genome [33]. The RdRP is indispensable for the viral replication cycle; without functional RdRP, RHDV cannot replicate its genome, resulting in the inability to produce infectious viral particles [34]. Given its crucial role, RdRP serves as a promising target for antiviral drug development. Inhibitors of RdRP activity have the potential to disrupt viral replication and serve as therapeutics against RHDV and related viruses [35]. This combined understanding of the functions of VPg and RdRP provides valuable insights into the replication mechanisms of RHDV and informs strategies for antiviral intervention.

Several studies reported the Immunomodulatory and antiviral effects of the black cumin seed [20, 36–38]. The active components found in Nigella sativa seeds mainly consist of volatile oils and alkaloids, which are primarily associated with various biological activities. Among these, the volatile oil constituents include nigellone, thymoquinone, thymohydroquinone, dithymoquinone, thymol, carvacrol, α- and β-pinene, D-limonene, D-citronellol, p-cymene, t-anethole, 4-terpineol, and longifolene [39, 40]. Noteworthy alkaloids encompass isoquinoline (nigellicimine and nigellicimine n-oxide) and pyrazol (nigellidine and nigellicine.(Thymoquinone is recognised as the principal bioactive compound, demonstrating a wide array of therapeutic properties [41].

In this study, before conducting molecular docking simulations, the structure of the VPg protein was predicted using computational modeling tools. The quality of the predicted VPg structure was evaluated using the PROCHECK server, ensuring that subsequent molecular docking simulations were based on reliable protein structures. This validation step is consistent with best practices in structural biology and computational drug discovery. Comparatively, similar studies have utilized computational modeling to predict protein structures and validate their quality. For instance, a study by [42] employed homology modeling to predict the structure of SARS coronavirus main protease, which was subsequently validated using Ramachandran plots and other structural assessment tools.

Molecular docking simulations were conducted to elucidate the binding interactions between selected compounds from Nigella sativa and the viral proteins, VPg and RdRP. The results demonstrated robust binding of all tested ligands to both proteins, with varying degrees of binding affinity. Notably, compounds such as nigellidine, nigellimine, and dithymoquinone exhibited high binding affinities towards both VPg and RdRP proteins, suggesting their potential as effective inhibitors of RHDV replication. Comparing these findings with similar studies [43], investigated the inhibitory effects of natural compounds against the Zika virus NS2B-NS3 protease through molecular docking simulations. Their results identified several compounds with strong binding affinities and antiviral potential.

Analysis of binding interactions between the ligands and viral proteins revealed intricate molecular mechanisms underlying their inhibitory effects. Pyrazole derivatives like nigellidine and nigellimine, as well as alkaloid derivatives such as nigellimine, formed multiple hydrogen bonds and other non-covalent interactions with the active sites of VPg and RdRP proteins. Similarly, volatile oils from Nigella sativa displayed diverse binding interactions, including hydrogen bonding and hydrophobic interactions, contributing to their strong binding affinities towards the viral proteins. Studies by [44] and [45] have explored the binding interactions of natural compounds with viral proteins using molecular docking. Their findings underscore the importance of hydrogen bonding and hydrophobic interactions in mediating the antiviral effects of natural compounds, consistent with the observations in the current study.

ADMET screening provided insights into the pharmacokinetic properties of the selected compounds, assessing parameters such as molecular weight, lipophilicity, and bioavailability. Ligands from Nigella sativa demonstrated favorable drug-likeness properties, adhering to Lipinski’s Rule of Five and exhibiting desirable pharmacokinetic profiles. Notably, compounds like thymohydroquinone and nigellidine displayed high lipophilicity, suggesting their potential for efficient tissue penetration and distribution. Studies by [46] and [47] have conducted similar ADMET screenings of natural compounds with antiviral potential. Their findings corroborate the favorable pharmacokinetic profiles observed in the current study, indicating the suitability of natural compounds from Nigella sativa as potential antiviral agents.

The MD results point to genuinely steady complexes. After the expected settling period, backbone RMSD traces flatten and stay there, while the ligand heavy-atom RMSD wobbles around the docked pose rather than drifting away (Fig. 9A–B). Radii of gyration are essentially flat as well—lower for VPg, higher for RdRP—so there is no sign of global compaction or swelling over 100 ns (Fig. 9C). Hydrogen-bond counts fluctuate but do not erode with time (Fig. 9D). In combination, these are the hallmarks of pose retention seen in polymerase–inhibitor simulations in other systems [22, 23].

The residue-level view supports the same story. Bound trajectories show lower RMSF in pocket-lining segments for both targets when set against the apo references (Fig. 10). In RdRP, the dampening extends around the canonical motifs, which is where conformational flexibility is often coupled to catalysis; tightening these regions is therefore consistent with reduced access to productive motions [10, 48–50]. For VPg, the reduction in local mobility maps onto surfaces implicated in priming/host-factor engagement, again matching the intended mode of action [10, 48].

Energetically, the MM-GBSA analysis ranks the four complexes in a way that agrees with docking (Fig. 11A). The component breakdown is typical for polymerase pockets—favorable van der Waals and electrostatic terms partially offset by polar solvation, with a modest non-polar contribution (Fig. 11B)—and is in line with how MM-GBSA behaves when evaluated over equilibrated frames for ligands of comparable size [24, 25]. While end-point methods are approximate, using many snapshots from the production window, as done here, generally improves relative ranking and helps connect persistent contacts to their energetic weight [24, 25].

PCA adds a complementary, dynamic view. In the PC1–PC2 plane, both proteins sample tighter regions in the bound state than in the apo reference (Fig. 12A–B), and porcupine readouts show smaller displacements near the pockets (Fig. 12C–D). Cosine-content values are low once equilibration is removed (Fig. 12E), indicating that these modes reflect equilibrated motions rather than diffusion-like drift [26, 27]. The simplest interpretation is that both ligands dampen “breathing” motions around the binding sites—precisely the sort of conformational narrowing that can disfavor catalysis or partner binding in RdRP and VPg [10, 48–50].

Finally, the choice of *Nigella sativa* constituents is supported by outside work. Several reports describe immunomodulatory and antiviral actions for this phytochemical space, with thymoquinone/dithymoquinone and related alkaloids repeatedly highlighted [51–56]. Against that backdrop, our MD/MM-GBSA/PCA results make a coherent case: nigellidine and dithymoquinone form stable complexes with VPg and RdRP, maintain favorable interaction energetics, and reduce motions in functionally sensitive regions. These findings strengthen the docking/ADMET ranking and outline specific, testable hypotheses for follow-up assays in RHDV models.

## Conclusion

This study employed computational methods to explore the inhibitory effects of compounds derived from Nigella sativa on Rabbit Hemorrhagic Disease Virus (RHDV) replication mechanisms, focusing on the viral proteins VPg and RdRP. Molecular docking simulations revealed robust binding interactions between selected compounds and these viral proteins, suggesting their potential as effective inhibitors of RHDV replication. Compounds such as nigellidine, nigellimine, and dithymoquinone were identified as top candidates based on strong predicted affinities and complementary hydrogen-bonding, hydrophobic, and other non-covalent contacts in the active pockets, alongside drug-likeness and lipophilicity supportive of tissue distribution. Follow-up MD (100 ns) showed steady backbone RMSD, retained ligand poses, and flat radii of gyration, while RMSF mapped reduced flexibility in pocket-lining segments (polymerase motifs in RdRP; cap-mimic/host-factor regions in VPg). MM-GBSA yielded favorable ΔG_bind values with van der Waals and electrostatic terms dominating and polar solvation partly offsetting, and PCA revealed narrowed conformational sampling and damped “breathing” motions in functionally sensitive regions. Together, these dynamic and energetic readouts substantiate the docking ranks and provide a mechanistic rationale to prioritise nigellidine and dithymoquinone for experimental validation toward antivirals targeting RHDV and related caliciviruses. This study provides valuable insights for the development of novel antiviral agents targeting RHDV and related viruses. Further experimental validation of these computational predictions will include in vivo evaluation in a rabbit model to confirm efficacy and safety, paving the way for the development of effective treatments for RHDV infection, addressing an important public health concern.

## Supplementary Information

Supplementary Material 1.

## Data Availability

Data is provided within the manuscript and available from the authors upon reasonable request from the corresponding author.
