# Supplementary figures and images for: Molecular docking insights of Nigella sativa compounds as potential antiviral inhibitory agents against the replication-machinery proteins VPg and RdRP in rabbit hemorrhagic disease virus (RHDV)
Source: Virol J. 2025 Nov 25;22:389. doi: 10.1186/s12985-025-03007-y (PMC12667127; doi:10.1186/s12985-025-03007-y)

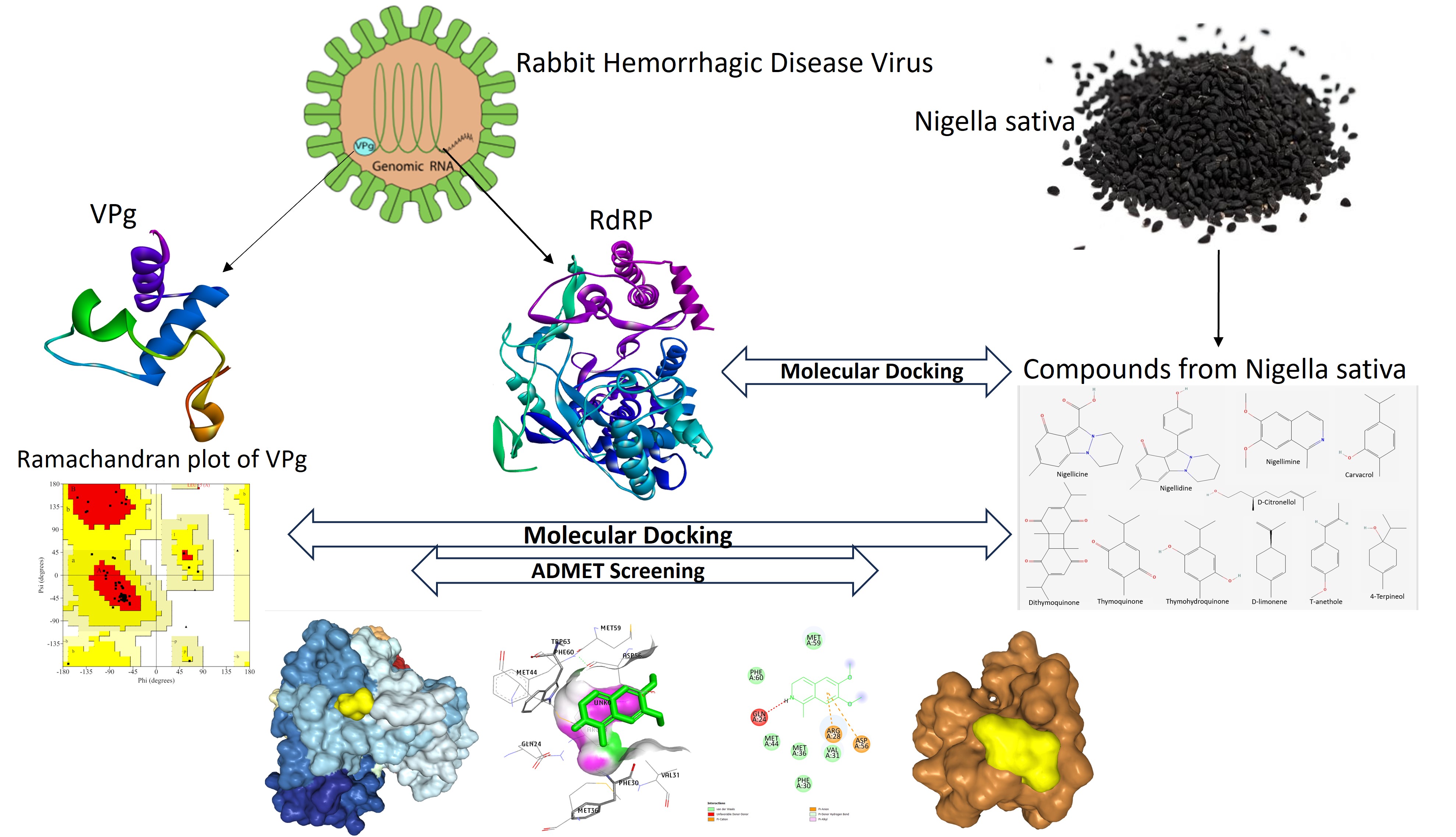

Supplement: Supplementary file 1 — Supplementary Material 1. [file 12985_2025_3007_MOESM1_ESM.jpg]
